# Fluorescence-Guided Surgery and Biopsy in Gliomas with an Exoscope System

**DOI:** 10.1155/2014/207974

**Published:** 2014-05-21

**Authors:** José Piquer, Jose L. Llácer, Vicente Rovira, Pedro Riesgo, Ruben Rodriguez, Antonio Cremades

**Affiliations:** ^1^Department of Neurosurgery, Hospital de la Ribera, Carretera Corbera km1, 46600 Alzira, Valencia, Spain; ^2^Department of Pathology, Hospital de la Ribera, Alzira, Spain

## Abstract

*Background*. The introduction of fluorescence-guided resection allows a better identification of tumor tissue and its more radical resection. We describe our experience with a modified exoscope to detect 5 ALA-induced fluorescence in neuronavigation-guided brain surgery or biopsy of malignant brain tumors. *Methods*. Thirty-eight patients with a suspected preoperative diagnosis of high-grade astrocytoma were included. We used a neuronavigation device and a high-definition exoscope system with a built-in filter to detect 5-ALA fluorescence in all cases. Thirty patients underwent craniotomy with tumor resection and 8 underwent frameless stereotactic brain biopsy. *Results*. Histopathological diagnosis confirmed the presence of high-grade gliomas in 34 patients. Total resection was achieved in 23 cases and subtotal in 7. No relevant complications related to the administration of 5-ALA were detected. *Conclusions*. The use of the exoscope in 5-ALA fluorescence-guided tumor surgery has twofold implications: during brain tumor surgery it can be considered a valuable tool to achieve a more radical resection of the lesion, and when applied to a biopsy of a suspected brain high-grade glioma, it decreases the possibility of a negative biopsy.

## 1. Introduction

High-grade gliomas represent the majority of adult malignant brain tumors and include grade III anaplastic astrocytoma (AA), anaplastic oligodendroglioma (AO), mixed anaplastic oligoastrocytoma (AOA), and grade IV glioblastoma multiforme (GBM) [1]. The best treatment of these tumors is extensive surgical resection, when it is possible, accompanied by chemotherapy and radiotherapy [2–4]. However, there are also cases in which biopsy is the best option [5].

Because of the infiltrative nature of these tumors, complete resection is a complex neurosurgical procedure, regardless of the location of the lesion. Several methods have been introduced to help achieve the maximum cytoreductive treatment. Recent developments, including brain mapping, neuronavigation, intraoperative ultrasound, magnetic resonance imaging, and fluorescence techniques [6, 7], now allow multimodal approaches. Under the suspicious of a high-grade lesion, when a brain biopsy is indicated, the stereotactic frame biopsy is still considered the gold standard technique for its precise localization [8], specially in cases of deep seated tumors. However, technological development in the neurosurgical practice has increased the number of centers in which brain biopsies are usually obtained by frameless magnetic resonance imaging- (MRI-) guided neuronavigation. Either way, the percentage of biopsies with inconclusive pathologic diagnosis varies between 7 and 15% in relation to nontumor or necrotic areas. Thus, even if a pathologist is available to study the sample at the time of the surgery, it is difficult to ensure that it will be useful for diagnosis [9].

It has been described that 5-ALA fluorescence helps to visualize tumor tissue in real-time during surgery, and most of the studies have been performed using fluorescence microscopy [10–13] or, much less frequently, an endoscope [14]. Both neurosurgical microscopes and neuroendoscopes may be modified to detect 5-ALA-induced fluorescence. This can be achieved by implementing a system to switch between white and blue light and installing an observation filter (440 nm) between the surgical field and microscope/endoscope. This can be done with an exoscope as well, which is a system that consists of a tubular telescope connected to a camera and a high definition monitor.

In this report we describe our experience in the treatment of high-grade gliomas using 5-ALA fluorescence-guided exoscopy, as an alternative or complement to the use of another optical device, in order to obtain the highest level of tumor resection or to confirm the adequacy of the specimen obtained during biopsy procedures.

## 2. Methods

The present study, approved by the Ethics Committee for Clinical Research of our institution, included 38 patients with preoperative suspicion of brain high-grade astrocytoma in neuroimaging techniques. Thirty patients underwent tumor resection and 8 underwent frameless stereotactic biopsy at the Department of Neurosurgery at Hospital Universitario de la Ribera (Alzira, Spain) between December 2012 and January 2014.

All patients received an oral dose of 20 mgr/Kg of body weight of 5-ALA 5-6 hours prior to the surgical procedure and were submitted to the standard protocols for low exposure to sunlight or artificial light. In all cases, clinical data were collected from electronic and paper medical records, including the suspected clinical diagnosis, the definitive histopathological diagnosis, and any systemic or neurological morbidity (progression of neurological deficit).

### 2.1. Preoperative Procedures

A brain 1.5-T MRI was performed as conventional preoperative study of the lesion in all cases. For those patients harboring tumors in proximity to eloquent areas (specially primary motor and sensory cortices, and primary language areas), a functional MRI was also performed. A Contrast T1-weighted MRI sequence in combination with a StealthStation Surgical Navigation System (Medtronic, Inc., Minneapolis, MI, USA) was used for preoperative planning and intraoperative neuronavigation of tumor resection or frameless stereotactic biopsy. All lesions were topographically classified according to Shinoda et al. [15]. Neurophysiologic monitoring by transcranial or cortical stimulation for motor-evoked potentials was performed in tumor resection cases in which the tumor was close to primary motor areas, and when necessary, the central groove was located by median nerve somatosensory-evoked potentials.

### 2.2. Surgical Procedure

When tumor resection was the goal, the routine procedure consisted of a tailored craniotomy according to the location of the lesion, followed with maximal tumor resection by using neuronavigation, 5-ALA fluorescence, and the appropriate fluorescence filter mounted in an exoscopy system. In those areas, where the tumor tissue looked red or pink under fluorescence, resection was carried out by alternating white and blue light in the exoscope. In patients with tumors close to eloquent areas, the resection stopped according to anatomical and neuronavigation limits, together with changes in neurophysiological monitoring indicating that an eloquent area could be compromised, even though remaining of some fluorescence tumor tissue was encountered. But in those with tumors not related to a particular eloquent area, the resection stopped when 5-ALA fluorescence was not visible, in conjunction to neuronavigation guidance of tumor limits. During surgery, several samples exhibiting different fluorescence intensities were obtained for tissue analysis. Fluorescence intensity was subjectively graded during surgery by both the surgeon and the assistant using a scale from 1 to 5 (5 being the highest intensity). In order to assess the extent of the resection, postoperative control MRI was performed in all craniotomy cases within the first 72 hours after surgery. Complete resection was considered when no enhancement was seen at the tumor cavity on T1-weighted imaging.

In frameless stereotactic biopsy cases we used the Navigus System (Medtronic), in combination with 5-ALA fluorescence and the exoscopy system. The biopsy was performed under general anesthesia and with the head fixed with the three-point Mayfield device. Automatic burr-hole insertion of 11 mm diameter was performed in the selected entry area, and, after assembling the biopsy system, a referenced needle of 1.2 mm diameter was introduced to reach the target. From 5 to 7 tumor samples of 1 to 2 mm size were obtained from each of the patients; the presence or absence of fluorescence was observed, and the samples were sent to histopathological examination.

### 2.3. Exoscope Description

**Figure 1:**
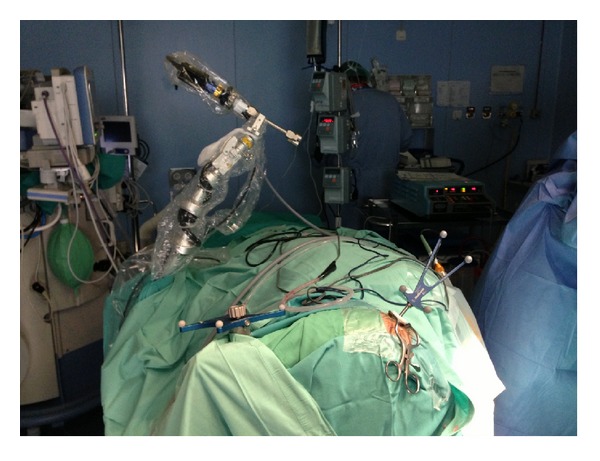
View of high-definition exoscope-assisted system in neuronavigation-guided biopsy.

For fluorescence detection, a high-definition exoscope system (HD-Xoscope, HDXO-SCOPE, Karl Storz Endoscopy, Tuttlingen, Germany) was used, including a specially developed autoclavable rigid lens telescope and a fiber optic light source channel (Figure 1). This system implements a 5-ALA filter blocking the excitation light while the fluorescence signal from the 5-ALA (380–450 nm) could pass through, along with normal white light. Upon excitation with blue light (lambda = 400–410 nm), protoporphyrin IX, the 5-ALA metabolite accumulated in tumor cells, is strongly fluorescent (peak kappa = 635 nm) and can be viewed in a specifically prepared neurosurgical optical system. Fluorescence emission may be classified as intense (solid) red fluorescence, corresponding to tumor tissue, or appear blue in the case of normal tissue not accumulating protoporphyrin IX, which reflects blue-violet light. The telescope is held in position by a pneumatic endoscope holder and the system is complemented with a 3-chip sterilizable high-definition digital camera, with optical zoom and focus features, and a video display with a medical grade 23” high-definition video monitor.

## 3. Results

The age of patients who underwent craniotomy (Table 1) was between 21 and 78 years with a mean age of 52 years. Eleven patients were women and 19 were men. The age range in patients who underwent biopsy (Table 2) was between 44 and 76 years with a mean age of 62.6 years. Four of these patients were men and 4 women.

In the craniotomy group, MRI evaluation indicated that total resection was achieved in 23 cases and subtotal resection in 7 patients. Progression of hemiparesis was observed in three patients after surgery, with subsequent partial recovery in two of them and transient dysphasia in one patient. Perioperative mortality occurred in a 79-year-old patient due to liver failure. In the biopsy group, no complications were seen. Minor adverse events related to the administration of 5-ALA were observed: a slight increase in serum GGT in one case and thrombocytopenia in two cases. However, no serious complications were registered in any of the groups.

Histopathological results in the craniotomy group reported three patients with metastases and 27 with high-grade gliomas: 23 with glioblastoma multiforme and two with oligodendroglioma and anaplastic astrocytoma in the remaining two of them. The mean fluorescence intensity, according to the subjective assessment of the surgeon and the assistant on a 1–5 scale, was 3.9 in the GBM group (IQR = 3.5–4.5), 3 (IQR = 2.5–3.5) in the cases of anaplastic astrocytoma, and 4 (IQR = 3.5–4.5) for the oligodendrogliomas. Of the three metastases, one showed a level 4 of fluorescence. On the other hand, histopathological results in the biopsy group revealed Glioblastoma Multiforme in 6 cases, one case with a central nervous system lymphoma and another one with an anaplastic astrocytoma. In this group, 5 to 7 samples were taken from each patient, with clear variations in the fluorescence intensity in relation to the tissue area obtained (either tumor cells or necrosis or peritumoral area). The intraoperative biopsy showed no fluorescence at all in the patient with central nervous system lymphoma.

When analyzing the tissue samples of both groups the most fluorescent areas observed intraoperatively corresponded to tumor, according to the histological results. Thus, when positive fluorescence was observed at the surgical site or in the frameless biopsy sample, the existence of tumor was confirmed in all cases (100%). The first 14 surgeries were performed without the 5-ALA filter blocked to the exoscope. In five of the craniotomy rest cases and in three biopsy cases, operated with the 5-ALA blocked filter, the histological findings of presence or absence of tumor with the presence or absence of fluorescence (red or blue) is correlated. Tumor tissue or infiltration areas were easily identified by their bright red or pink color, while nontumor area reflected the blue light. In those patients where no fluorescence was observed (blue), the absence of tumor was confirmed in 62% of them (5 patients); necrosis was observed in other two, and only in one (12%) was the existence of brain tissue with a small infiltrative tumor area revealed. Although the sample is very small, this would result in a sensitivity of 73% and a specificity of 100%, or, what is the same, in a negative predictive value of only 63% but a positive predictive value of 100%.

## 4. Discussion 

**Figure 2:**
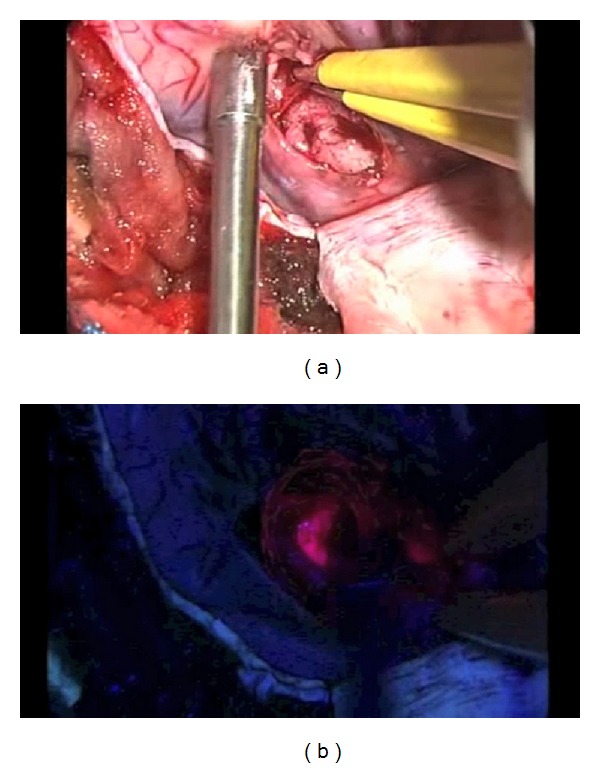
Glioblastoma multiforme surgical view. (a) Tumor and infiltration area without fluorescence. (b) Tumor and infiltration area under 5-ALA fluorescence.

Various authors have described that fluorescence-guided tumor resection increases the rate of complete excision of high-grade gliomas, without significantly increasing morbidity [4, 12, 16–22]. 5-ALA fluorescence allows for tumor “visualization” in real-time during surgery, including diffuse infiltration areas in which tumor cells are mixed with normal parenchyma, so the malignant tissue can be easily identified [23, 24] (Figures 2(a) and 2(b)). The study performed by Stummer et al. [13] validated the use of 5-ALA fluorescence to guide the resection of high-grade gliomas using a microscope, describing complete resection in 65% of patients treated with 5-ALA compared to 36% without 5-ALA, with a 6-month disease-free survival rate and common adverse effects between both groups.

In our study, of the 27 high-grade gliomas (23 GBM and 4 anaplastic gliomas) in the craniotomy group, total resection was accomplished in 79.3% of the cases, with clinical morbidity in three patients, these results being very similar to those observed in larger series.

Analogous to the microscope, our high-definition exoscope has been adapted to detect 5-ALA-induced fluorescence. The filter combination/specification is exactly the same as the one found in surgical microscopes from other manufacturers, sharing similar image properties. The exoscope obtains high quality images with a wide field and a target distance of 200 mm. This wide range allows it to be set far from the surgical field, and the instruments can be used with fluoroscopy, which is impossible with traditional neuroendoscopes. The exoscope is also helpful for visualizing deeply located lesions. On the other hand the exoscope is less heavy and expensive when compared with a microscope. It also allows both the surgeon and the assistant to adopt an ergonomic position, making it possible to perform surgery with “four hands,” or to switch continuously from a microscopic to a macroscopic view during microsurgery, while the whole team has access to the same images [25]. Furthermore, allocating a microscope to detect fluorescence in the samples of guided biopsy involves using a disproportionate remedy and prevents using it at the same time in other open surgical procedure where it is most needed.

**Figure 3:**
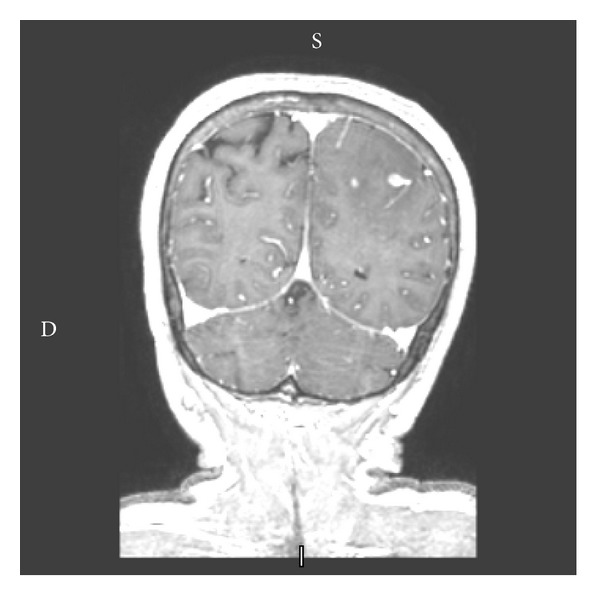
Coronal T1-weighted MR image with gadolinium showing a partially enhancing lesion.

**Figure 4:**
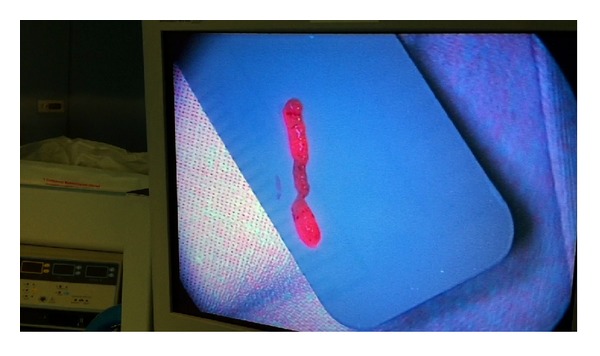
Sample obtained from tumor area in a neuronavigation-guided biopsy.

In frameless stereotactic biopsy, despite the high accuracy of neuronavigation systems in relation to the target point chosen, the samples obtained are not always useful for pathological diagnosis. Biopsies can be nondiagnostic in 2–15% of cases. Several causes for this result have been described, such as small lesion size, deep location, presence of necrosis, poorly differentiated tumors, lack of contrast enhancement, and deep tumor location [9]. This means that in many centers biopsies should be examined intraoperatively by the pathologist, verifying that the sample contains tumor areas, with a consequent increase in operative time and the need for the presence of staff who can examine them. However, Shooman et al. [5] question the utility of intraoperative neuropathological assessment. They observe that intraoperative neuropathology rarely influences the procedures that are being performed. Furthermore, false-positive results left open the possibility that biopsy might be ceased prematurely despite an ultimately negative sample. In addition, a false-negative result may necessitate continuation of an ultimately fruitless procedure despite the acquisition of ample material. Since 5-ALA is a safe medication (there are few reported adverse reactions) and easy to administer, and as a result of the experience obtained from fluorescence-guided tumor resection, we think that its use would increase the diagnostic yield of brain biopsies. Panciani et al. [26] assessed the reliability of a multimodal strategy based on 5-ALA and neuronavigation and the combined approach represented the best sensitivity to tumor tissue. In the present series, all the fluorescent samples obtained within the tumor boundaries, and according to the neuronavigation, were positive for high-grade glioma in the pathological examination. 5-ALA-induced fluorescence showed high sensitivity for the assessment of malignant glioma and, interestingly, in cases of confirmed metastasis a high intraoperative fluorescence level was appreciated in the samples, even though they usually exhibit an inhomogeneous fluorescence pattern, as reported by other authors [27]. Inflammatory cells and reactive astrocytes in the peritumoral area may appear fluorescent. In contrast, necrotic areas can appear negative despite being intratumoral. Despite our limited series of patients, during biopsy procedures in high-grade gliomas, when relying on neuronavigation accuracy for identification of the tumor target, combined with positive fluorescence in the samples collected, the possibility that they would not be adequate for histopathological examination is minimal (Figures 3 and 4).

## 5. Conclusion

The high-definition exoscope system, modified to detect 5-ALA-induced fluorescence, is useful both for guided surgery of suspected high-grade gliomas and frameless MRI stereotactic biopsies, acting as a valuable tool to achieve more radical resections in brain tumor surgeries and decreasing the chances of negative biopsies. Further advantages, compared to other systems, include a lower cost and simple and manageable mobilization and transportation, as well as allowing the surgeon to perform his act in a more ergonomic position and lessening the surgeon's fatigue.

## Figures and Tables

**Table 1:** Clinical characteristics of tumor resection cases.

Case	Age	HPD	ALA I	L	Lobe	Resection grade	PND	Systemic CPL
1	40	AA	4	B-1-1	Left frontal	Subtotal	No	PTE
2	50	GBM	4	B-0-1	Left frontal	Total	No	No
3	61	GBM	5	A-0-0	Right parietotemporal	Total	No	TCP
4	43	GBM	4	A-0-0	Right temporal	Total	No	No
5	59	GBM	5	B-1-1	Left frontal	Subtotal	No	No
6	79	GBM	2	A-1-0	Right temporal	Total	No	TCP
7	28	OLG GIII	1	A-1-0	Right frontal	Total	No	No
8	45	OLG GIII	3	A-1-0	Right frontal	Total	No	No
9	50	AA	2	B-1-1	Left temporal	Subtotal	No	No
10	66	GBM	2	A-0-0	Right temporal	Total	No	No
11	44	GBM	4	B-1-1	Left temporal	Total	No	No
12	55	GBM	4	B-1-1	Left temporal	Subtotal	No	GGT
13	56	GBM	5	A-1-0	Right parietotemporal	Total	No	PTE
14	76	MET	5	B-1-1	Left temporal	Total	No	No
15	63	GBM	1	A-0-0	Right parietal	Total	No	PTE
16	68	MET	5	B-1-1	Left frontal	Total	HEM P	No
17	20	GBM	4	A-0-1	Left parietooccipital	Total	No	No
18	49	GBM	5	A-1-0	Right frontotemporal	Subtotal	HEM P	No
19	58	MET	4	A-1-0	Right frontal	Total	HEM P	No
20	66	GBM	1	A-1-0	Right temporal	Total	No	No
21	72	GBM	5	A-0-1	Right occipital	Subtotal	No	No
22	66	GBM	4	A-0-0	Right parietal	Total	No	No
23	64	GBM	4	A-0-0	Right parietal	Total	No	No
24	57	GBM	5	A-0-0	Right parietal	Subtotal	No	No
25	21	GBM	4	A-0-1	Left parietooccipital	Total	No	No
26	70	GBM	4	A-1-0	Right temporal	Total	No	No
27	23	GBM	5	B-1-1	Left frontal	Total	No	No
28	44	GBM	5	B-1-1	Left parietal	Total	No	No
29	34	GBM	4	A-1-0	Right frontal	Total	No	No
30	59	GBM	4	A-1-0	Right parietotemporal	Total	No	No

HPD: histopathological diagnosis; AA: anaplastic astrocytoma; GBM: glioblastoma multiforme; OLG: oligodendroglioma; G: grade, MET: metastasis; L: tumor location (Shinoda); location-size-eloquence of adjacent parenchyma; PND: postoperative neurological deficit; HEM P: hemiparesis progression; CPL: complications; PTE: pulmonary thromboembolism; TCP: thrombocytopenia; GGT: gamma glutamyl transferase.

**Table 2:** Clinical characteristics of tumor biopsy cases.

Case	Age	HPD	ALA I	L	Lobe	PND	Systemic CPL
1	76	GBM	3	C-0-0	Basal ganglia	No	No
2	44	GBM	4	C-1-0	Corpus callosum	No	No
3	70	GBM	4	B-0-1	Left temporal	No	No
4	60	GBM	5	B-0-1	Left temporal	No	No
5	50	GBM	4	A-1-1	Right frontoparietal	No	No
6	62	LINFOMA	1	C-1-1	Bilateral hemispheric	No	No
7	70	GBM	4	B-0-1	Left frontal rolandic area	No	No
8	69	GBM	3	A-0-1	Right parietofrontal	No	No

HPD: histopathological diagnosis; GBM: glioblastoma multiforme; L: tumor location (Shinoda); location-size-eloquence of adjacent parenchyma; PND: postoperative neurological deficit; CPL: complications.
